# Is the Addition of Sublingual Melatonin to Omeprazole Superior to Omeprazole Alone in the Management of Gastroesophageal Reflux Disease Symptoms: A Clinical Trial

**DOI:** 10.5152/tjg.2023.23021

**Published:** 2023-12-01

**Authors:** Habib Malekpour, Amin Noori, Saeed Abdi, Mohammad Abbasinazari, Arash Mahboubi, Mahdiye Abiyar Ghamsari

**Affiliations:** 1 Department of Gastroenterology and Hepatology, Imam Hossein Hospital, Shahid Beheshti University of Medical Sciences, Tehran, Iran; 2 Department of Clinical Pharmacy, Shahid Beheshti University of Medical Sciences Faculty of Pharmacy, Tehran, Iran; 3 Department of Gastroenterology and Hepatology, Gastroenterology and Liver Diseases Research Center, Research Institute for Gastroenterology and Liver Diseases Ayatollah Taleghani Hospital, Shahid Beheshti University of Medical Sciences Tehran, Iran; 4 Department of Pharmaceutics, Pharmaceutical Sciences Research Center, Shahid Beheshti University of Medical Sciences Faculty of Pharmacy, Tehran, Iran

**Keywords:** Melatonin, omeprazole, GERD, sublingual, quality of life

## Abstract

**Background/Aims:**

Proton pump inhibitors are frequently used to treat gastroesophageal reflux disease, but their effect is restricted. The present study aimed to investigate whether the addition of sublingual melatonin to omeprazole was effective in the treatment of gastro gastroesophageal reflux disease symptoms.

**Materials and Methods:**

This was a randomized double-blind clinical trial. A total of 78 patients with gastro gastroesophageal reflux disease were randomly allocated to either omeprazole 20 mg/d plus sublingual melatonin (3 mg/d) or omeprazole 20 mg/d plus placebo for 4 weeks. The selected patients had histories of heartburn and regurgitation and a score ≤32 on the Frequency Scale for the Symptoms of gastroesophageal reflux disease (FSSG). The outcome measures for the assessment of treatment efficacy were heartburn, epigastric pain and the Frequency Scale for the Symptoms of gastroesophageal reflux disease score. Safety and quality of life were evaluated in the patients as the secondary outcomes too.

**Results:**

Seventy-two out of 78 eligible patients completed this trial (35 in the melatonin group and 37 in the placebo group). Heartburn, epigastric pain, and Frequency Scale for the Symptoms of gastroesophageal reflux disease score declined significantly in the melatonin group compared to the placebo group (*P* = .04, *P* = .03, and *P* = .0001, respectively). Moreover, the quality of life score was significantly higher in the melatonin group compared with the placebo group (*P = *.0001). Adverse events were similarly observed in the 2 groups (*P = *.55), and there were no serious adverse events.

**Conclusion:**

The combination of sublingual melatonin (3 mg/day) with omeprazole (20 mg/day) may be more effective than omeprazole (20 mg/day) alone in the treatment of gastroesophageal reflux disease.

**Main Points**

- Acid suppression, especially proton pump inhibitors, is widely accepted as the cornerstone of medical treatment of gastroesophageal reflux disease.
- It is estimated that around 30% of patients with diagnosis of gastroesophageal reflux disease are refractory to proton pump inhibitors so attempt for evaluation of new medication for gastroesophageal reflux disease pharmacotherapy is necessary.
- Melatonin has been used for the treatment of gastroesophageal reflux disease symptoms successfully but there is a concern regarding poor bioavailability of melatonin from gastrointestinal tract. So in the present trial, the aim was to investigate whether the addition of sublingual melatonin to omeprazole was effective in the treatment of gastroesophageal reflux disease symptoms.

## Introduction

Gastroesophageal reflux disease (GERD) is one of the most common digestive disorders worldwide. The prevalence of this disease is estimated at 8%-33% in all age groups in both males and females; it is also one of the costliest digestive disorders.^1^ The most common digestive symptoms of GERD are heartburn and regurgitation, while its atypical symptoms include chest pain, chronic cough, pseudo-influenza symptoms, and sinusitis.^2, 3^

Today, proton pump inhibitors (PPIs) are the most important group of drugs for reducing stomach acid secretion. They are used for the treatment of many upper digestive disorders.^4^ Proton pump inhibitors exert the most significant therapeutic effects against GERD. In nearly 30% of patients with a diagnosis of GERD, these drugs cannot improve their condition. The failure of PPI treatment is an important challenge in the control and management of GERD. The reasons for this failure include mutations in the H^+^/K^+^ ATPase coding gene, presence of a polymorphism in the cytochrome p4502c19 enzyme, nocturnal acid breakthrough, delayed stomach discharge.^5^ Also considering pH testing, patients with diagnosis of GERD are classified into 3 groups: those with unusual acid reflux scores (nonerosive reflux disease), those with usual acid reflux scores and a direct relation of symptom with reflux (acid-sensitive esophagus), and those with normal reflux scores and no symptom related to reflux events (functional heartburn). Data have been reported that the patients with nonerosive GERD treated better with PPIs therapy than those in the other 2 groups.^6^

Melatonin is used to improve sleep disorders in many countries. It is secreted in the pineal gland to control sleep patterns and in the enterochromaffin cells to improve the digestive system motility.^7^ It has been postulated that low melatonin levels are effective in the aggregation of GERD symptoms, as melatonin leads to diminished stomach acid secretion and strengthens the lower esophageal sphincter.^8^

Melatonin leads to the stimulation of melatonin type 2 (MT2) receptors in the duodenal enterocyte. Stimulation of MT2 leads to increase of bicarbonate secretion; accordingly, they provide protection of the duodenal epithelium against stomach acids. Besides, stimulation of these receptors reduces stomach acid secretion.^9^ Melatonin deficiency leads to increased nitric metabolites, along with the diminished antioxidant activity of enzymes.^10^ Additionally, it can inhibit biosynthetic nitric oxide. The transient lower esophageal sphincter relaxation is one of the main mechanisms of GERD, where nitric acid plays a key role.^11^

Melatonin, by inhibiting oxygen activation through neutralization of oxygen, functions as an antioxidant and free radical scavenger and consequently, exerts gastroprotective effects.^12^ A study by Kandil et al^13^ indicated that oral melatonin alone could reduce GERD symptoms (heartburn and epigastric pain). Moreover, in a study by Klupińsk et al^14^ melatonin administration improved the symptoms of functional dyspepsia. In previous studies, the oral tablet form of melatonin was used for the treatment of upper gastrointestinal disorders, with low bioavailability (about 15%) in the gastrointestinal tract.^15^ Attempts to increase the bioavailability of melatonin seem rational to improve the management of different diseases. Therefore, the present study aimed to investigate the efficacy, safety, and quality of life (QOL) of patients with GERD, who received sublingual melatonin, melatonin plus omeprazole, or placebo to control their symptoms.

## Materials and Methods

This double-blind randomized clinical trial was approved by the ethics committees of nursing, midwifery, and pharmacy schools of Shahid Beheshti University of Medical Sciences (code: IR.SBMU.PHARMACY.REC.1400.168) and registered in the Iranian Registry of Clinical Trials (code: IRCT20121021011192N12). Before initiating the clinical stages, melatonin and placebo were prepared and packaged. Melatonin (3 mg) was prepared as sublingual tablets (Vana DarouGostar Pharmaceutical Company, Iran), and the placebo was prepared at the pharmaceutical laboratory of the Shahid Beheshti University of Medical Sciences, with the same appearance as sublingual melatonin, according to the main drug formulation without melatonin.

The clinical stages of this study were initiated on May 1, 2022 and continued until October 1, 2022. The patients were randomly assigned to either the melatonin or placebo group based on the table of random numbers. The patients, physician, and examiner were blinded to the drug or placebo during the trial. The patients in the melatonin group received 20 mg of omeprazole in the morning before breakfast plus 3 mg of sublingual melatonin at night. In the placebo group, the patients received 20 mg of omeprazole in the morning before breakfast plus melatonin placebo sublingually at night.

This study was performed on patients with mild to moderate symptoms of GERD, who were referred to the gastroenterology clinic of Shahid Beheshti Hospital as outpatients. At baseline, the patients were explained about the study and the course of treatment, and volunteers signed written informed consent forms for participation in this study. The inclusion criteria were as follows: diagnosis of GERD; age above 18 years; having complaints of heartburn and or regurgitation 2 days or more per week; and a score ≤32 on the Frequency Scale for the Symptoms of GERD (FSSG). In this questionnaire, the symptoms associated with the severity of disease included heartburn, throat burning sensation, esophageal dysphagia, chest pain, and voice hoarseness, which were evaluated in 20 items and scored based on a 0-4 Likert scale, with higher scores indicating more severe symptoms.^16^

The exclusion criteria were as follows: history of sensitivity to omeprazole or melatonin; patients with reflux symptoms with diagnosis of peptic ulcer and duodenal ulcer on endoscopy; pregnant or breastfeeding women; current use of drugs causing transient lower esophageal sphincter relaxation (e.g., calcium channel blockers and nitrates); patients with Child-Pugh C cirrhosis; patients with severe renal damage (Acute Kidney Injury Network [AKIN] classification stage 3); shift workers; patients using medications with major interactions with omeprazole or melatonin (e.g., fluvoxamine); patients using alternative or traditional medicines concurrently; and patients with severe symptoms (FSSG score >32).

After selecting eligible volunteers for the study, demographic characteristics, including sex, age, smoking habits, and body mass index (BMI), were recorded. Also, before treatment, FSSG and QOL scores were calculated for the patients. To evaluate QOL, the validated Persian version of Mayo-gastroesophageal reflux questionnaire was employed. This questionnaire was completed before and after the study to evaluate the QOL of the patients. It contained 25 items, with the scores ranging from 25 (the lowest QOL) to 175 (the highest QOL)^16, 17^; in other words, higher scores represented higher QOL.

At baseline, the patients received the training needed to optimize their lifestyle for controlling GERD symptoms (e.g., consuming food at low volumes, but higher frequencies per day, not consuming alcohol, not wearing tight clothes, and avoiding lower esophageal sphincter loosening foods). Next, patients in the 2 groups took the medication or placebo for 4 weeks. After 4 weeks, they would return to the gastroenterology clinic for reexamination. In the second visit, they completed the FSSG and GERQ. During 4 weeks, the patients were contacted at least once a week to inquire about their adherence to medications, as well as possible adverse drug reactions (ADRs). If ADRs were attributed to the drug or placebo, the patient was recommended to stop taking the drug or placebo and was excluded from the study.

### Statistical Analysis

For data analysis, the Statistical Package for Social Sciences (SPSS) version 26.0 (IBM Corp.; Armonk, NY, USA) was used. For data comparison, mean ± SD was measured. The minimum level of statistical significance was set at *P* ≤ .05. The normal distribution of parameters was evaluated using Kolmogorov–Smirnov test. For comparison of the placebo and melatonin groups, samples *t*-test, Mann–Whitney test, and Pearson’s chi-square test were utilized. To calculate the sample size required for this study, the FSSG was used as the main index. For the FSSG score to reach 10 in the melatonin group and 12 in the placebo group within 1 month,^18^ at least 35 patients were required for each group considering a SD of 3, an alpha level of 0.05, and a beta value of 80%.

## Results

**Figure 1.**
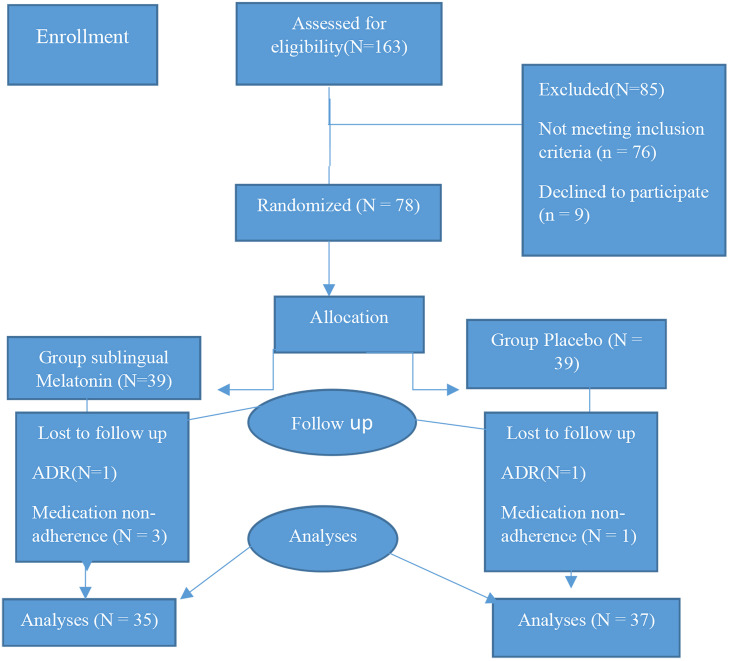
Flow diagram of the study.

In this study (5 months), 163 patients with GERD symptoms, who were referred to the clinic and willing to participate in this research, were included; however, 85 patients were removed based on the exclusion criteria. Out of 78 patients included in the study, 4 from the melatonin group and 2 from the placebo group were excluded during the study due to ADRs of the drug and lack of drug adherence. In the final analysis, 35 and 37 patients in the melatonin and placebo groups were evaluated, respectively. Figure 1 presents the process of patient recruitment in this study. Also, Table 1 reports and compares the demographic characteristics of the patients in the 2 groups. Statistical analyses indicated no significant differences in terms of sex, BMI, and smoking habit (*P* = .81, .43, and .76, respectively). However, the mean age of the melatonin group was significantly lower than that of the placebo group (*P* = .0001).

Table 2 outlines the number of patients with epigastric pain and heartburn in each group before and after the study. Statistical analyses showed that the frequency of patients with epigastric pain and heartburn did not differ significantly before treatment (*P* = .59 and *P* = .48, respectively). However, at the end of the study, the number of patients with heartburn and epigastric pain reduced in the melatonin group compared to the placebo group, and this reduction was statistically significant (*P* = .04 and *P* = .03, respectively).

The scores of FSSG and GERQ questionnaires were determined and compared between the melatonin and placebo groups before and after the study. The FSSG scores were not significantly different between the 2 groups before treatment (*P* = .30). After 4 weeks and at the end of the study, post-treatment FSSG score reduced in both groups; this reduction was significant in both melatonin and placebo groups compared to the baseline (*P* = .001 and *P* = .0001, respectively). The FSSG score was lower in the melatonin group compared to the placebo group, and the difference was statistically significant (*P* = .0001). In other words, the melatonin group showed a greater FSSG score reduction compared to the placebo group.

Additionally, the GERQ scores of the 2 groups were determined before and after the study, as shown in Table 2. Statistical analysis revealed that the value of this index was not significantly different between the 2 groups at baseline (*P* = .41). After 4 weeks, the GERQ score increased more considerably in the melatonin group compared to the placebo group, and the difference was statistically significant (*P* = .0001). The main ADR was drowsiness in the 2 groups (5 cases in melatonin and 3 cases in placebo group). Also nausea, vomiting, mouth dryness and headache have been reported in some patients (less than 3 cases in any side effects). Although the incidence of ADRs was higher in the melatonin group, no significant difference was found between the 2 groups (*P* = .55).

## Discussion

Although PPIs are the first-line treatment for GERD, considering the high prevalence of GERD symptoms in Iran (10%-14.9%) and around the world (13%), besides the high rates of disease relapse and chronicity, a high percentage of patients do not respond to this type of treatment, thereby highlighting the need for new compounds with minimal side effects.^19-21^ In recent years, studies have evaluated the role of melatonin in controlling the symptoms of GERD. In this regard, Kandil et al^13^ conducted a study on 36 patients with GERD, who were assigned into 4 groups of 9. The first group received omeprazole (20 mg/d), the second group received melatonin (3 mg/d), the third group received omeprazole plus melatonin concurrently, and the fourth group only received the placebo. After 4 weeks, control of symptoms, such as heartburn and epigastric pain, was considerable in the omeprazole, melatonin, and omeprazole plus melatonin groups compared to the baseline. The efficacy of omeprazole alone was greater than melatonin, and the concurrent use of melatonin and omeprazole was more effective than other groups. Nevertheless, due to the small sample size, further trials are recommended.

In a meta-analysis by Bang et al^22^ on studies evaluating the efficacy of melatonin in the treatment of GERD, after reviewing medical databases, including EMBASE, Cochrane, and PubMed, the efficacy of oral melatonin tablets in the management of GERD symptoms was reported. Since the bioavailability of oral melatonin tablets is below 15% in different studies,^23, 24^ in this trial, to achieve a higher blood level of melatonin and desirable therapeutic effects, the sublingual form of this drug was used. Considering the overlap between the symptoms of upper digestive system diseases, including functional dyspepsia, a reliable questionnaire (FSSG) was used in this study for the initial diagnosis and precise grading of patients with GERD. This questionnaire can be used for evaluating the severity of disease and response to treatment, as well as diagnosis by the healthcare team and physicians.^25^

Numerous studies have reported a relationship between plasma melatonin level and its efficacy in the treatment of upper digestive disorders. A study by Chojnacki et al^10^ examining the effect of melatonin on *Helicobacter pylori*-induced dyspepsia, found that melatonin level was significantly lower in the group of *H. pylori* plus dyspepsia (*P* < .001); after 6 months of treatment with melatonin, the extent of improvement in dyspepsia symptoms was 47% in the placebo group and 84.3% in the treatment group.

Since previous studies have suggested that the sublingual form of melatonin can establish higher plasma levels,^26^ it might pose a risk to the significant alleviation of digestive symptoms compared to its oral form with lower bioavailability. Accordingly, in this study, the sublingual form of melatonin was used. To the best of our knowledge, no clinical study has yet investigated the effects of sublingual melatonin on controlling GERD symptoms. The present results based on FSSG showed that melatonin significantly contributed to the alleviation of GERD symptoms compared to the placebo (*P* = .001). Nevertheless, in the placebo group, the symptoms diminished significantly compared to the baseline, which is most probably due to treatment with omeprazole.

Finally, the results of the current study indicated the greater effect of melatonin plus omeprazole versus omeprazole alone in the management of GERD symptoms, such as heartburn, epigastric pain, and FSSG score. Moreover, the simultaneous use of melatonin and omeprazole compared to omeprazole alone was associated with greater improvements in the QOL of patients after 4 weeks. In a review study by Bang et al,^22^ the use of melatonin was associated with decreased sleep disorders, which would indirectly reduce the symptoms of GERD.^21^ Besides, Gurges et al^27^ reported that GERD is mostly associated with sleep disorders in patients. Since sleep improvement is one of the scoring indices in GERQ, the melatonin group was expected to obtain better scores compared to the placebo group, which is consistent with the results of the present study. Additionally, the current study showed that sublingual melatonin was not associated with serious ADRs and was well-tolerated by the patients; the similar incidence rates of side effects in the 2 groups confirm this finding.

Although this is the first study examining and comparing the effects of sublingual melatonin addition to omeprazole versus omeprazole alone, it has some limitations. Unlike some previous research, assessments in this study only included subjective parameters, while more objective parameters, such as manometry, pH monitoring, and measurement of melatonin concentration, were not examined because of financial and time constraints to identify the relationship between plasma melatonin level and management of symptoms for confirmation of the results. In our trial, the age was different significantly between the 2 groups. This may be related to the sample size of the trial. Randomization error (variability, imprecision) can be overcome by increasing the sample size. Although a positive effect of sublingual melatonin has been observed in our trial, more sample sizes recommend strongly in future trials. Future studies are highly recommended to address these shortcomings.

## Figures and Tables

**Table 1.** Demographic Characteristics of the Patients in Sublingual Melatonin and Placebo Group (n = 72)

	Sublingual Melatonin (n = 35)	Placebo (n = 37)	*P*
Mean age (years) (±SD)	38.18 ± 13.28	35.62 ± 11.2	<.0001
Sex			
Female	19	19	.81
Male	16	18	
Body mass index (kg/m^2^) (±SD)	24. 53 ± 3.18	23.01 ± 3.58	.43
Smoker			
No	28	31	.76
Yes	7	6	

**Table 2.** Baseline and Secondary Outcomes in Sublingual Melatonin and Placebo Group (n = 72)

	Sublingual Melatonin Group (n = 35)	Placebo Group (n = 37)	*P*
Before treatment heartburn	32	31	.48
Before treatment epigastric pain	27	26	.59
After treatment heartburn	4	13	.04
After treatment epigastric pain	4	9	.03
Before treatment FSSG	23.48 ± 5.88	22.32 ± 4.76	.30
After treatment FSSG	10.02 ± 2.43	14.97 ± 3.28	.0001
Before treatment quality of life	91.94 ± 13.04	94.86 ± 17.07	.41
After treatment quality of life	128.28 ± 10.20	115.10 ± 15.77	.0001

FSSG, Frequency Scale for the Symptoms of gastroesophageal reflux disease.
